# Delivering an efficient and effective support group for patients with implantable cardioverter-defibrillators (ICDs): patient perspectives of key concerns and predictors of inclination to attend

**DOI:** 10.1186/s12913-021-06735-3

**Published:** 2021-07-27

**Authors:** Kathryn Murray, Kelly Buttigieg, Michelle Todd, Vicky McKechnie

**Affiliations:** 1 Department of Clinical Health Psychology and Neuropsychology, Imperial College Healthcare NHS Trust, London, UK; 2 West London NHS Trust, London, UK; 3 Department of Cardiac Investigations, Imperial College Healthcare NHS Trust & West London NHS Trust, London, UK

**Keywords:** Implantable cardioverter-defibrillator, ICD concerns, Support group, Qualitative

## Abstract

**Background:**

A subset of patients experience psychological distress following insertion of an implantable cardioverter-defibrillator (ICD) and ICD support groups are recommended, however access to these groups is limited. This study aimed: to gauge a better understanding of patients’ key ICD-related concerns; to determine patient interest in a support group and topics deemed helpful to address in a support group; and to examine factors which affect patient inclination to attend.

**Methods:**

One hundred and thirty nine patients completed the ICD Patient Concerns Questionnaire – Brief (ICDC-B) and a semi-structured survey. Non-parametric tests were used to examine associations and differences in the quantitative data. Qualitative data were analysed using thematic analysis.

**Results:**

42% of respondents said they would attend a support group and inclination to attend was associated with higher ICD concerns and a shorter time since implant. Topics considered important to address in a group were information about heart conditions and devices, the impact of an ICD on daily life and coping with fear of shocks.

**Conclusion:**

We concluded that there is interest in further support amongst many patients and that ICD support groups may be delivered efficiently by targeting patients who have higher levels of ICD concerns and within the first few years after implant.

## Introduction and aims

The implantable cardioverter-defibrillator (ICD) is a highly effective treatment for preventing arrhythmic death and rates of implantation have increased significantly over the last three decades [1]. Despite the life-saving benefits of ICD treatment, the literature suggests that a number of patients experience significant psychological distress following implant [2, 3]. Several challenges may contribute to ICD-related distress, including: the diagnosis and management of a potentially life-threatening cardiac condition; reliance on a device; an altered sense of self; and living with the potential for shock and mortality [4, 5]. A recent review of patient perspectives of living with an ICD highlighted the importance of addressing patients’ key concerns [6]. Patient dissatisfaction was associated with a misalignment in priorities, with healthcare professionals over-emphasising clinical aspects rather than their patients’ actual concerns, such as quality of life and normal functioning post-implantation. Understanding the aspects of living with an ICD that patients find most challenging and offering targeted support which addresses these issues is therefore imperative to improving patient care.

Studies have shown that combined interventions using education and cognitive behavioural therapy (CBT), in parallel to cardiac rehabilitation exercise, can reduce anxiety, depression and ICD concerns [7–9], however access to cardiac psychology services is variable. Given that resources in most clinical services are already severely stretched, it is essential that ICD groups are delivered as efficiently as possible. This study aimed to enable the efficient delivery of effective ICD support groups, by clarifying patient perspectives of key challenges, patient interest in a support group, topics deemed helpful to address in a support group and factors which affect patient inclination to attend a support group.

## Methods

All methods were carried out in accordance with relevant guidelines and regulations.

### Setting and intervention

During a four month period (May to September 2017), patients with ICDs attending a routine outpatient appointment at a pacing clinic were invited to complete questionnaires assessing ICD-related concerns and patient experiences of living with an ICD. When patients arrived for their clinic appointment the receptionist invited patients to complete the survey whilst waiting for their appointment. Any patient with a functioning device was eligible for the study, with the exception of the following exclusion criteria: high levels of patient distress based on clinical judgement, and cognitive or language barriers to completion of the questionnaires. The clinic is located within an acute trust of inner London but national referrals are also accepted. Patients recruited to the study were of mixed socio-economic status.

### Outcome measures

#### The ICD patient concerns questionnaire – brief version (ICDC-B)

ICD-related concerns were assessed, using the ICDC-B [10] which is an eight-item validated measure adapted from a 20-item questionnaire [11]. Patients rate the items (e.g. ‘I am worried about my ICD firing’) on a five-point Likert scale from 0 (not at all) to 4 (very much so). There are no standardized cut offs and higher total scores (range 0–32) indicate greater device-related concerns. The ICDC-B has good internal consistency, with a Cronbach’s α = 0.91) [10].

#### Patient surveys

A brief semi-structured survey was designed for the purpose of this study, specifically to obtain patient demographics, cardiac diagnosis, ICD type (primary or secondary prevention) and time since implantation, as well as gauging more information about patient perspectives of living with an ICD, and patient interest in attending a support group. All answers were according to patient self-report. The survey included open-ended questions and the option to select up to ten topics that they would value being included in an ICD support group. These topics were chosen based on areas of concern identified in the literature.

### Data analysis

Quantitative data from the questionnaires were analysed using the statistical package, SPSS for Windows 10. Non-parametric tests were used as appropriate. Qualitative data from the surveys were collated and analysed using thematic analysis [12]. Two researchers independently coded the responses and the initial review indicated that the researchers were in agreement on approximately 90% of initial codes. Divergence of initial codes for the remaining responses was resolved by further review and discussion with a third researcher. The three researchers then discussed and agreed meaningful themes, which were checked against the initial codes and the overall dataset, and amended where necessary. The final themes were subsequently organised into overarching domains.

#### Ethical considerations

The study was submitted to Information Governance and registered with the Trust as a service evaluation project. All aspects of the study protocol were approved by the Information Governance department of the Trust. The survey included an information paragraph explaining that the researchers were hoping to set up a support group for patients with ICDs and that the purpose of the research was to learn more from patients about their experiences of living with an ICD and their views about a support group. Patients were informed that their participation was voluntary and that their responses would remain confidential.

## Results

Surveys were given to patients attending a routine appointment at 30 pacing clinics between 24th May and 12^th^September 2017. All eligible patients attending these clinics were invited to complete the questionnaire.??? patients who were not considered appropriate because they appeared too distressed to concentrate or there was a language barrier. A total of 146 questionnaires were returned; 8 patients declined to participate for the following reasons: patient preference [4], time constraints [2], difficulty understanding the questionnaire [1] forgetting their glasses [1].

Of the 146 returned surveys, two were omitted due to language limitations, five due to insufficient completion, and two because the patient indicated that they did not have a functioning ICD, leaving a total of 139 surveys in the analyses. Demographic and clinical characteristics are shown in Table 1.

**Table 1:** Demographic and clinical characteristics of participants

Age	Median = 66 years (range 35–87)
Gender	106 M (76%); 33 F (24%)
ICD indication	63 (45%) primary prevention
	47 (34%) secondary prevention
	16 (11%) don’t know; 13 (9%) missing
Time since implant	Median = 40 months (IQR 12–204)
Diagnosis	Ischemic cardiomyopathy: 52 (37%)
	Non-ischemic cardiomyopathy (structural genetic abnormalities):35 (25%)
	Non–ischemic cardiomyopathy (with normal heart structure):8 (6%)
	Multiple diagnoses/not possible to discern from answer: 16 (12%)
	Heart failure: 11 (8%)
	Missing – 17 (12%)

### Key ICD concerns

Total scores on the ICDC-B ranged from 0 to 32 (median = 6, IQR = 2–10). Total ICDC-B score was not-normally distributed, with a skewness of 1.5. There were no demographic or clinical predictors of ICD concerns: Spearman correlations, Mann.

Whitney U tests and Kruskal Wallis tests indicated that there were no correlations between age or time since implant with ICDC- B total and no associations between gender, diagnosis and ICD indication with ICDC-B total.

### Qualitative analysis

One hundred and thirty one patients responded to the open-ended questions in the patient survey. Thematic analysis of these responses yielded eight themes across two domains. Each domain is described below with illustrative quotations of each theme (Table 2).

**Table 2:** Domains, themes and illustrative quotations

Domain and theme	Frequency	Illustrative Quotation
**Emotional impact of ICD**		
Sense of gratitude, safety and reassurance	42	*‘Massive impact – grateful’.* *‘Knowing I have my ICD has given me peace of mind to know I have a life saving device as my guardian angel’.* *‘Without doubt it has removed the fear of a fatal heart incident’*
Increased anxiety and sense of vulnerability	15	*‘The reality of having a heart condition is difficult enough, the thought of the ICD shocking me!’* *‘It has increased my consciousness of my vulnerability’.*
Adjustment over time	10	*‘At first you notice the weight but now I forgot it is even there’.* *‘While after fitting feeling of resenting ICD for a week or two’.*
**Functional and Role Changes**		
Self-identity and role changes	5	*‘Losing my core role, reduction in income and quality of life. Loss of control over my working life’.* *‘I feel it has taken part of my freedom away’.*
Improved functioning and quality of life	18	*‘I have improved health and well-being, less breathlessness, less tired, increased energy’.* *‘It has improved my general wellbeing’.* *‘Improved my stamina. I get tired but not exhausted anymore.’*
Physical discomfort	15	*‘The size of it- BIG. It is still sore and uncomfortable. Restricts my movements and I keep ‘tweaking’ it when I move my arm in certain ways’.* *‘My sleep is poor due to discomfort of the ICD’*
Practical adjustments	37	*‘Restriction with enjoying certain activities,* i.e. *grand-children. Reduces your activity level’.* *‘No football, no cricket’.* *‘Airports, can’t go through scanning machines, extra time needed to go through.’*
Minimal consequences	63	*‘Hasn’t impacted on my life, got on with life’.* *‘I don’t really notice it much (forget it’s in)’* *‘I feel no difference.’*

### Emotional adjustment

Over half of the patients who responded to the open-ended questions in the survey referred to the emotional impact of having an ICD and many described a sense of gratitude that the device saved their life and/or a sense of reassurance and safety gained by having an ICD. Conversely, a smaller group of patients described increased anxiety since having their ICD, often due to a fear of the device firing, or an increased sense of vulnerability due to greater awareness of their heart condition. Some patients reflected on an adjustment process, involving initial difficult thoughts and feelings about their ICD which resolved with time.

### Functional and role changes

Nearly half of the respondents indicated that their ICD had not had a significant impact on their lives, or they described minimal negative consequences. A sizeable group also talked about positive changes to their health and overall well-being since having their ICD, such as fewer symptoms and increased energy. Conversely, some patients said that their physical functioning had declined due to discomfort, for example, initial healing pain and more persistent pain which also interfered with sleep, although this tended to be amongst patients who were in the earlier stages of recovery after ICD insertion.

Approximately a third of patients said that their ICD had affected their daily lives and the practical adjustments included minor disturbances, as well as more profound effects on valued activities. Moreover, a minority of patients described changes that had affected their self-identity and core roles, with one patient reporting that he was unable to continue working.

### Intention to attend a support group

Fifty eight (42%) of the participants who responded said that they would attend a support group for patients with ICDs. Patients who indicated that they would attend had their ICD implanted more recently (median = 45 months) than those who indicated that they would not attend a support group (median = 63 months, *p* < 0.03). Patients who would attend reported higher levels of ICD concerns (median ICDC total = 6) than those who would not attend a support group (median ICDC total = 4.7, *p* < 0.02).

### Support group topics

All patients were asked to indicate which topics would be helpful to address in a support group, even if they were unable to attend themselves and 116 patients completed these questions. The frequency of endorsements for each topic are given in Table 3.

**Table 3:** Frequency of endorsements for support group topics

Topic	Number of endorsements
Information about heart conditions	72 (62%)
Impact of having an ICD on daily life	63 (54%)
Information about ICDs and how they function	63 (54%)
Coping with fear of shocks	62 (53%)
The emotional impact of having an ICD	47 (41%)
How our psychological reaction affects adjustment	35 (30%)
Impact on sex life	27 (23%)
Impact on relationships and changed roles	25 (22%)
Body image concerns	24 (21%)
Impact on self-image	21 (18%)

## Discussion

The first aim of this study was to explore patients’ key ICD concerns and perspectives of living with an ICD. The quantitative data indicated variable levels of ICD concerns with a median score on the ICDC-B comparable to that found in previous studies [13, 14]. The qualitative findings from the patient surveys provided further insight into patients’ key concerns and perspectives of living with an ICD. As found previously [15], this study demonstrated that ICDs are well accepted by many patients, with over half of the participants reporting that their ICD had minimal impact on their lives. Furthermore, some patients described improved emotional well-being, due to the reassurance and safety net acquired by having an ICD, or improved functioning, due to a reduction in cardiac symptoms. However, a sizable minority of participants described emotional difficulties, namely increased anxiety, self-identity and role changes, practical adjustments and physical discomfort. These challenges are consistent with difficulties described by patients with ICDs in previous studies [5], but healthcare providers often do not have the time or resources to address these concerns as they have to prioritise the clinical aspects of patients’ care during medical consultations [6]. The provision of specialised support which addresses these broader aspects of living with an ICD is likely to significantly improve patient care.

Amongst the patients who described feeling more anxious since having their ICD, fear of shocks was a common concern. Many studies have shown that anxiety about the potential for shocks is highly prevalent amongst ICD patients [16] and that concerns about shock, rather than a shock itself, determine psychological adjustment [10]. Providing psychological support to help patients manage this anticipatory anxiety is likely to be another crucial element of an ICD support group.

Inclination to attend a group was also associated with a shorter time since ICD implant, which suggests that support groups may be most valued by patients within the first few years after implant. Indeed, a recent systematic review suggested that patients often experience uncertainty and higher levels of distress in the initial phase after implant, before they transition to an adjustment phase, involving adaptation and acceptance [6]. Another study found that informing patients whilst they were still in hospital about the option of participating an ICD support group, was associated with higher levels of attendance [17]. These findings suggest that patients may most need and be most likely to attend a support group if it is offered in the earlier stages after their implant.

The most popular topics to be included in a support group were information about heart conditions, the impact of an ICD on daily life, information about ICD devices and coping with fear of shocks. It is vital that patients are provided with an appropriate space to ask questions and develop accurate beliefs about their heart condition and device. A poor understanding of one’s device predicts poor psychological adjustment [18] and it is well documented that inaccurate health beliefs are associated with adjustment difficulties [19]. Patients indicating that they would also highly value the opportunity to discuss the broader impact of their ICD on their daily life and how to cope with fear of shocks further indicates that these are pertinent issues that patients with ICDs require further support with.

The clinical implications of these findings are that healthcare providers should consider providing support groups to patients with higher levels of ICD-related distress and within the first few years after implant. In addition to increasing efficacy by targeting support towards patients who are most likely to attend, we would suggest that offering group support remotely via videotherapy may also be helpful, particularly given the current restrictions of the COVID-19 pandemic, the high financial impact and logistical demands of offering face-to-face group programmes, and the reality that many patients travel significant distances to pacing clinics.

We note that there are some limitations to this study. Firstly, it was a small-scale study carried out in a busy, clinical setting, and therefore only one measure of ICD-related distress was examined. It would be valuable for future research to examine whether other previously identified risk factors for ICD-related distress, such as history of ICD shock, ICD knowledge, ICD acceptance, ethnicity, and specific type of ICD device, also relate to patient interest in an ICD support group. Another limitation is that demographic and clinical data were not collated for the eligible patients who declined to participate, which may reduce the generalisability of the findings. However, as only a small minority of eligible patients declined participation, we believe that our findings do reliably reflect the views and experiences of the majority of the ICD population.

## Conclusions

A significant minority of patients experience anxiety, functional and role changes. There is interest in further support amongst many patients and topics considered of most value are information about heart conditions and devices, the impact of an ICD on daily life and coping with fear of shocks. Patients with higher levels of ICD concerns and patients with ICDs implanted within the previous three years are most likely to attend these support groups. Healthcare providers could deliver ICD groups efficiently by targeting these patients.

## Data Availability

The datasets used and analysed during the current study are available from the corresponding author on reasonable request.
